# Continuous-flow analysis: the Auto-Analyzer

**DOI:** 10.1155/S146392468500030X

**Published:** 1985

**Authors:** C. Crandell

**Affiliations:** Spectra-Physics Inc. 3333 N. First Street San Jose California 95134 USA


Journal of Automatic Chemistry/*Journal of Clinical Laboratory Automation*, Vol. 7, No. 3 (Jul–Sep 1985), pp. 145–148

### Continuous-flow analysis: the Auto-Analyzer

**C. Crandell**
*Spectra-Physics, Inc., 3333 N. First Street, San Jose, California 95134, USA*

An increase in the work-load of clinical and analytical chemistry laboratories in the 1940s resulted in a drive towards automation. The repetitive nature of analyses, with the associated tedium, provided further incentive toward developing automated systems. To these ends, Skeggs conceived Continuous-Flow analysis (CFA), introduced commercially by the Technicon Instruments Corporation in 1957 in the form of an automated system under the name 'Auto-Analyzer' [1 and 2].

The term 'continuous flow' refers to the flow of liquid continually pumped through the system while it is being analysed for particular components. Samples, standards, and reagents are injected into this stream at regular intervals and a report is generated only after all injections have been made. In some cases a run may consist of as many as 120 injections.

The first CFA systems were modular in design and were used for large-volume tests such as serum glucose and blood-urea-nitrogen levels. A typical system used by the pharmaceutical industry is shown in figure 1. Simpler systems use a peristaltic pump to pass samples and reagents through a temperature-controlled reaction coil and into a colorimetric detector.

### Components

The modular nature of CFA allows parts to be switched as needed. A general CFA contains the following parts (see figure 1): a sampler which selects the item to be injected into the flow stream; a pump to keep the flow stream in motion; a separation or mixing device; a heat source to speed up reactions; a detector to monitor the concentration of a particular component; and a data handling/presentation device to perform calculations and report results.

The sampler is the device that holds the specimen-bearing vials. The specimens consist of vials of standards of known concentrations, to provide a calibration mechanism; analysis samples; reagents for mixing with samples and standards; blanks to flush the columns; and controls to monitor the calculation process. The sampling mechanism controls both volume and the delivery rate of the specimen in the circular tray, although some autosampling devices allow the user to program movement of the sampler mechanism to search for a specific sample located in the tray.

The rate of specimen delivery is governed mechanically by a 'programming cam'. As this cam rotates at a constant motor speed, valves are opened and closed, resulting in an alternating introduction of sample and solvent. The solvent is referred to as the 'wash' because it is used to flush remnants of the immediately preceding sample from the column to prevent or minimize its effects on succeeding samples. The ratio of the time the sample is being pumped to the time the solvent is being pumped is thus referred to as the 'sample-to-wash' ratio. The most common sample-to-wash ratio is 2:1, although ratios from 9:1 to 1:6 are possible. The specimen introduction rate is on the order of 40 to 60 samples per hour, but can go as high as 150 specimens/h.

The potential problem of sample diffusing into the solvent as it moves through the tubing and the column to the detector can be minimized by introducing a small air bubble in between injections of the different varieties of the specimen.

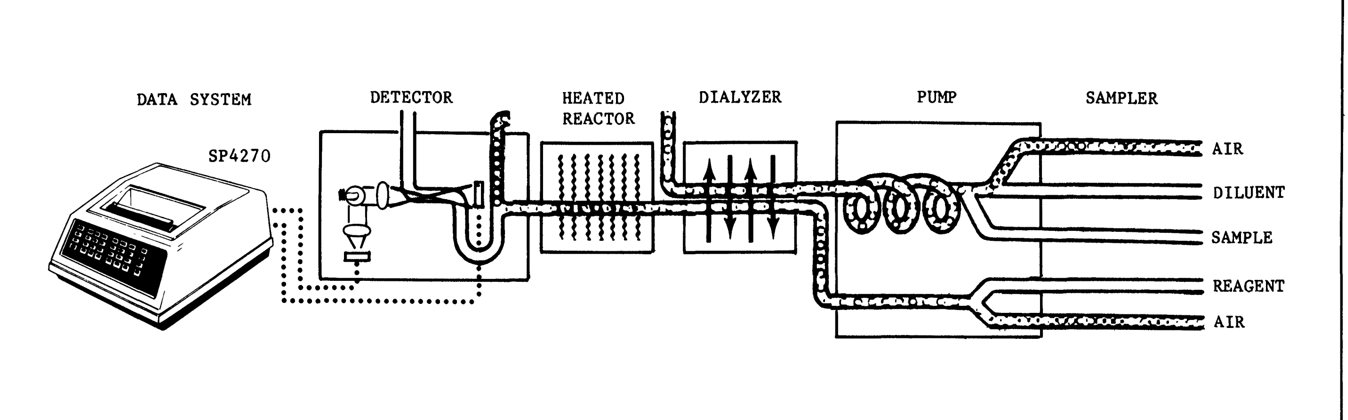

*Figure 1. Typical Auto-Analyzer and data system.*

Specimen identification is another area of consideration. Human error in the preparation of specimens, labelling containers and insertion into the sample tray can be minimized by automating the preparation process and by the use of bar codes and bar-code readers. It is estimated that the level of gross random errors can be reduced from 5% to 1% by automating sample preparation and identification techniques.

The pump serves to keep liquid moving through the Auto-Analyzer system. Pliable tubing is commonly used as a transmission medium, so thought must be given to the potential interaction between the specimen and the tubing, and, more importantly, to stretching of the tubing during runs.

The variation in the tubing affects the reproducibility of the results, and is one cause of drift. This real problem can be monitored by including controls (standards) along with the samples. During the calculation process, if the calculated concentration of one of the controls exceeds a defined tolerance about the known concentration, either the run can be repeated (possibly with new tubing), or allowances for this drift can be made in calculations. In the case of allowances, some form of computing capability is of obvious benefit.

If there is a need to separate one component from the specimen, a column or a dialyzer can be used. With a dialyzer, a semi-permeable membrane separates compounds of low molecular mass from those of high molecular mass. The object is to push as many specimens through the dialyzer as fast as possible to keep the sample throughput high. The result is that equilibration is never quite attained, so the resulting detected signal represents some fraction of the actual component. Reproducibility and accuracy therefore depend on the dialysis process occurring to the same extent for all standard and sample solutions.

When a chemical reaction occurs, provision must be made for it to occur as quickly and as completely as possible. The reaction could be between two reagents that produce a species of interest, or the species of interest forming a light-absorbing species. This may be done by providing an elevated temperature or a time delay. Since a time delay slows down the overall system, a heating mechanism is the method of choice to accelerate the reaction.

Any flow-through detector can monitor the concentration of the component of interest. The most commonly used is either a colorimetric detector, flame photometer, fluorometer, spectrophotometer, or atomic absorption flame photometer.

The simplest reporting device is the strip-chart recorder. This provides a trace which can be saved, but all the necessary calculations must be done by the user. When dealing with a 60–150 analyses run with a least squares multilevel calibration method, this can be a formidable task. Ideally, the reporting device should analyse the data automatically, perform all the necessary calculations and display the results in an understandable form. A BASIC-programmable computing integrator (like the Spectra-Physics SP4200 or SP4270) provides the necessary calculating and reporting flexibility.

### Auto-Analyzer problems solved by a computing integrator

Common Auto-Analyzer problems that a BASIC-programmable computing integrator can solve are: variation of report to meet specific laboratory's needs, variation of calculations to suit the analysis type; ability to correct for drift as the situation demands; ability to use controls to check calculations and modify calibration curves as necessary; and assignment of peaks in a realm where times of data are not unique identifiers.

The standard report is a concise tabulation of data and results. While this is acceptable in some cases, often the report must be printed out in a specific format. An individual report is shown for one of the samples making up an Auto-Analyzer run (figure 2), and a summary report (figure 3) is shown listing the information the researcher sees as most important for a series of samples. Note that the individual report also shows the coefficients of the calibration curve calculated via linear least squares fit (intercept and slope). Further, for this particular application, a test had to be made to see whether the calculated percent composition was within 2% absolute and 5% relative of a guarantee. It was, so a 'sample OKAY' message was included in the report. Using the graphing capability of the SP4200, a plot of the calibration data can be obtained (figure 4).

```
Lab #         Inspector#     Analyst      Date
**1603**      405-31         J. Jones     3/10/84

Method:       Nitrogen-CFAA

Instrument:   CFAAS

Parameters:   FILTER-660NM, CELL-15MM, WASH-60SEC, SAMPLE-120SEC

Standards     Standard Conc  Net Std Peak Ht   Intercept    Slope

    1             150 ppm         53.3            -7.3      0.404

    2             180 ppm         65.2

    3             200 ppm         72.5

    4             250 ppm         93.7

Sample Mass   Sample Dilution    Peak Ht   ppm Final Dilution

  0.3128          250 ml         74.83          203.5

Lab Sample#   Guarantee      % in original sample   (out of range msg)

 **1603**       16.00%          **16.26%**             Sample OKAY

```

*Figure 2. Individual sample report from Auto-Analyzer by SP4270 Computing Integrator.*

```

Method:         Nitrogen-CFAA

Instrument:     CFAAS

Parameters:     FILTER-660NM, CELL-15MM, WASH-60SEC, SAMPLE 120SEC

  Lab      Insp                                   Net
  Samp#    Samp#       Weight      Dilution     Peak Ht    Guar%     Orig%
  1603     405-31      0.3128        250         74.83     16.00     16.25
  1604     405-32      0.3135        250         75.83     16.00     16.41
  1605     405-33      0.3126        250         74.70     16.00     16.23
  1606     405-34      0.3101        250         79.08     16.00     17.24  <—
```

*Figure 3. Summary report from Auto-Analyzer by SP4270 Computing Integrator.*

Two problems that plague the Auto-Analyzer process are drift and carry-over. Drift is noticeable (figure 5) because over the course of a run (1 h or so) physical and electrical changes cause differences in the detected signals. Baseline drift can be dealt with within the computing integrator. More localized sources of drift can be mathematically dealt with by the use of controls, essentially samples of known concentrations used to monitor the detection and calculation processes. For instance, if two controls of identical concentration are used to 'bracket' a series of samples, a local drift contribution can be computed using the following formula:

$$H'_i = H_i - [t_i - t_0]^* [H_n - H_0]/[t_n - t_0] \quad (1)$$

$H'_i$ = corrected height of $i$th peak;
$H_i$ = measured height of $i$th peak;
$H_0$ = measured height of leading control;
$H_n$ = measured height of trailing control;
$t_0$ = retention time of leading control;
$t_i$ = retention time of $i$th peak;
$t_n$ = retention time of trailing control.

Carry-over is a common, but solvable problem caused by incomplete sample flushing. In this case, a set of three samples (a first of high concentration followed by two of low, identical concentrations) can be used to calculate a carry-over factor which is used to correct peak heights:

$$CO = [H_2 - H_3]/H_1 \quad (2)$$

$CO$ = carry-over factor;
$H_1$ = height of first carry-over standard peak;
$H_2$ = height of second carry-over standard peak;
$H_3$ = height of third carry-over standard peak.

This factor can now be used to correct the measured height:

$$H'_i = H_i - CO * H_{i-1}. \quad (3)$$

Whereas in most chromatographic runs peaks are caused by chemical compounds having unique retention times,

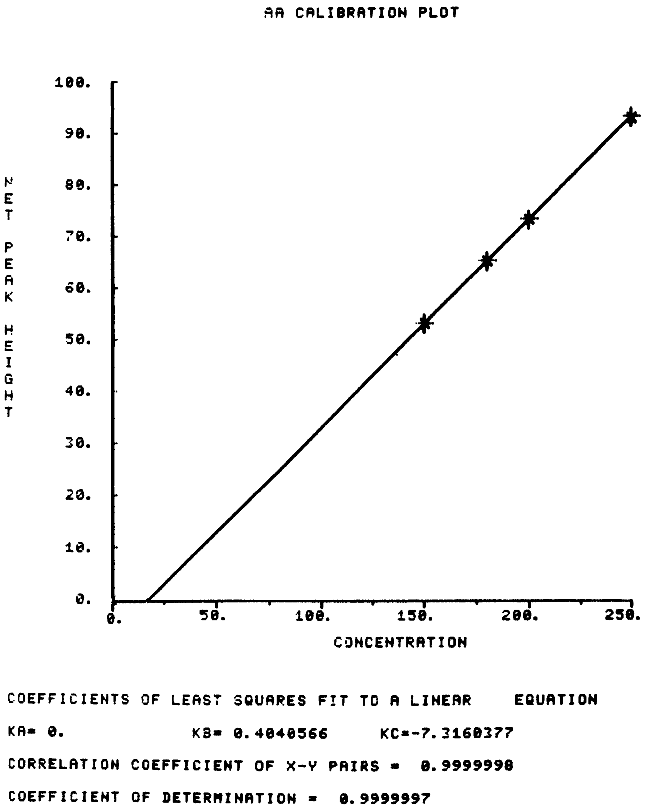

*Figure 4. Calibration plot generated by the SP4200 Computing Integrator.*

all that is known about retention times in an Auto-Analyzer run is what the difference in successive times is supposed to be, i.e. the cycle time, made up of sample and wash time. For the calculation process, it is important to identify correctly peaks as standards, carry-over, control, blanks, and samples, in order to calculate least squares curve, correct peak heights and sample concentration.

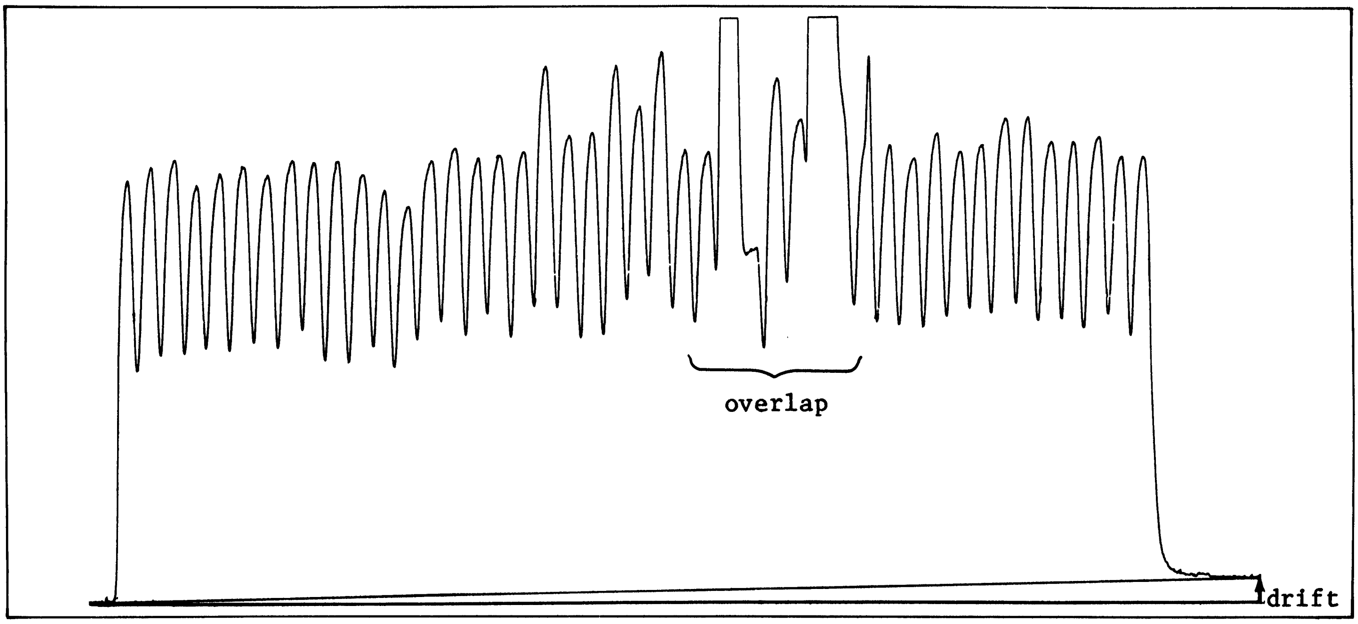

*Figure 5. Base-line drift caused by physical and electrical changes and overlap caused by peaks of high concentration.*

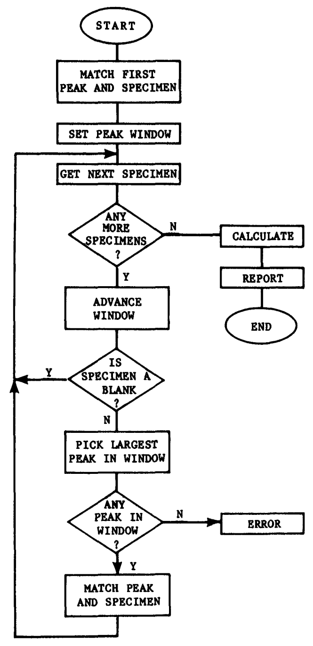

*Figure 6. Peak assignment algorithm.*

The simplest assignment process involves matching based on the information provided during run set-up.

There will be gaps caused by the presence of solvent blanks which may produce spurious peaks of low height and area. Besides these gaps, peaks of abnormally high concentration that significantly overlap neighboring peaks (see figure 5), may complicate the process of peak assignment and concentration calculation.

The peak assignment method of choice takes into account both the cycle time between peaks and the pending specimen type. The flowchart in figure 5 illustrates the assignment process.

### Examples

Depending on the needs of the user, the required program can be very simple, or very complicated. For example, one simple program uses a single point calibration (calibration peak and concentration selected by the user) to determine an RF value which is then applied to all peaks. It is a seven-line program, 166 bytes long.

A next step in complexity involves multi-level calibration capability, and uses least squares calibration curves. If a Spectra-Physics RX + Chip is present, the calibration curve can be plotted; the report notes sample name and concentration. This custom-designed program is 70 lines and 3000 bytes long, much of which is 'HELP' information.

Both of the above Auto-Analyzer programs assume that there is an exact correspondence between detected peaks and the number of standards and samples. This is not always the case – spurious peaks are sometimes detected; if sample concentrations are sufficiently high, peaks may significantly overlap; and factors like drift and carry-over must be taken into account. To handle all this requires another level of complexity. This program is being developed, but is not yet available. Being more complex than the previous two examples, this program will also be longer and require more memory; however; it will address the majority of features needed to handle an Auto-Analyzer run successfully.

### References

1. MacDonald, J. C., *Pharmaceutical Technology* (April 1978).
2. Tietz, N. W., *Fundamentals of Clinical Chemistry* (W. B. Saunders Co., 1976).

### 1985 EASTERN ANALYTICAL SYMPOSIUM

*19 to 22 November 1985 at the New York Penta, USA*

For the first time, the EAS will host a short course for chemistry undergraduates at its 1985 annual meeting in NYC. A course in analytical problem-solving will be offered free to chemistry majors. It is intended to introduce undergraduates to the opportunities available in the field of analytical chemistry. By participating, these students will learn that there are challenging problems that are not related to routine sample analysis. They will also be made aware of what the industry can offer to a graduate analyst.

The EAS is continuing its preparations for the exhibition of analytical instruments and services: nearly 150 of the 198 booths have now been reserved. A large number of exhibitor workshops have been proposed and will include topics on AA, automated sample preparation, computerized chemistry, particle sizing, robotics, ion-chromatography, FT-IR and many others. Technical programmes are also being prepared. There are plans for 44 sessions of invited papers, four sessions of contributed papers and 80 poster papers.

*More information from Harvey Gold, Department of Chemistry, University of Delaware, Newark, Delaware 19716, USA.*

